# Vitamin D Is Necessary for Murine Gastric Epithelial Homeostasis

**DOI:** 10.3390/biology10080705

**Published:** 2021-07-23

**Authors:** Ifrah Ismail Ali, Iltaf Shah, Sayed Marzouk, Sherif M. Karam, Asma Al Menhali

**Affiliations:** 1 Department of Biology, College of Science, United Arab Emirates University, Al Ain 15551, United Arab Emirates; 200540058@uaeu.ac.ae; 2 Department of Chemistry, College of Science, United Arab Emirates University, Al Ain 15551, United Arab Emirates; altafshah@uaeu.ac.ae (I.S.); sayedm@uaeu.ac.ae (S.M.); 3 Department of Anatomy, College of Medicine & Health Sciences, United Arab Emirates University, Al Ain 15551, United Arab Emirates; skaram@uaeu.ac.ae

**Keywords:** vitamin D receptor, vitamin D_3_, gastric epithelial homeostasis, cell proliferation, cell differentiation

## Abstract

**Simple Summary:**

The gastric epithelium comprises multiple cell types that undergo continuous renewal through controlled proliferation, differentiation and death. Several vitamins, such as vitamin D (VD), are known to contribute to tissue homeostasis and numerous studies have shown the importance of VD in different body organ systems. However, VD’s normal function in the stomach is understudied. To better understand the role of VD in the murine stomach, we initially confirmed the expression of VD receptors (VDR) in the stomach. Mice were fed a VD-deficient diet for 3 months. The results showed that the proton pump of acid-secreting parietal cells was downregulated in vitamin D-deficient mice and contributed to an abnormal gastric physiology. Moreover, this diet increased the gastrin hormone gene expression and increased gastric epithelial cell proliferation. These findings suggest essential biological roles for VDR and VD in gastric epithelial homeostasis. Future research will be required to explore this phenomenon in humans.

**Abstract:**

Unlike other organs, the importance of VD in a normal stomach is unknown. This study focuses on understanding the physiological role of vitamin D in gastric epithelial homeostasis. C57BL/6J mice were divided into three groups that were either fed a standard diet and kept in normal light/dark cycles (SDL), fed a standard diet but kept in the dark (SDD) or fed a vitamin D-deficient diet and kept in the dark (VDD). After 3 months, sera were collected to measure vitamin D levels by LC-MS/MS, gastric tissues were collected for immunohistochemical and gene expression analyses and gastric contents were collected to measure acid levels. The VDD group showed a significant decrease in the acid-secreting parietal cell-specific genes *Atp4a* and *Atp4b* when compared with the controls. This reduction was associated with an increased expression of an antral gastrin hormone. VDD gastric tissues also showed a high proliferation rate compared with SDL and SDD using an anti-BrdU antibody. This study indicates the requirement for normal vitamin D levels for proper parietal cell functions.

## 1. Introduction

Vitamins are nutritional factors required in small quantities that play important roles in human development, reproduction and tissue maintenance [1]. Although many vitamins are obtained through diet, several, such as vitamin D (VD) [1], are changed into their effective forms, which require further activation in the body. VD is universally known as a steroidal hormone because it has pro-steroids as a precursor. VD exists in two forms, VD_2_ (ergocalciferol) [2] and VD_3_ (cholecalciferol) [3]. Both VD_2_ and VD_3_ could be derived from food and dietary supplements; however, the primary source of cholecalciferol is the skin. 7-dehydrocholesterol, the precursor of VD_3_, is naturally found in epidermal cells. 7-dehydrocholesterol is converted into VD_3_ in the plasma membrane following exposure to ultraviolet B radiation from the sun. As VD_3_ reaches the extracellular fluid and then the blood, it is relocated to the liver after binding to a VD binding protein (DBP) [4]. Within the liver, the enzymes sterol 27-hydroxylase (CYP27A1) and 25-hydroxylase (CYP2R1) turn VD_3_ into 25-hydroxyvitamin D_3_ (25OHD_3_), which is then primarily translocated to the kidney via the DBP where it is hydroxylated further by 1α-hydroxylase (CYP27B1) to become the biologically active form, 1α,25-dihydroxyvitamin D_3_ (1α,25(OH)_2_D_3_). This active form is transported to multiple target tissues, including bone and the intestines, to perform its biological functions [5].

The VD receptor (VDR) is a nuclear receptor [6] and it belongs to receptor subfamily 1, group I and member 1 [7]. The function of the VD receptor is strongly dependent on its molecular nature and its particular structure. In most cases, when 1α,25(OH)_2_D_3_ is attached, the VDR will act as a ligand-dependent transcription factor and it will also allow the 1α,25(OH)_2_D_3_-VDR complex to interact with RXR. These 1α,25(OH)_2_D_3_-VDR-RXR heterodimers are carried to the nucleus where they bind to VD response elements (VDRE) in the promoter sequence of the VD target genes. Thus, the activated VDR directly induces coactivators and corepressors that regulate the transcription of target genes and translation of target proteins responsible for mediating VDR functions [8,9]. Target genes include Ca^2+^ channels and transient receptor potential vanilloid member 6 (*TRPV6*) [10], important in inducing a capsaicin-dependent apoptosis in gastric cancer cells [11]. 1α,25(OH)_2_D_3_ also regulates the transcription of the parathyroid hormone-like hormone (*Pthlh*) [12] and it is also involved in the activation of osteoclasts and the proliferation and differentiation of chondrocytes [13]. Moreover, via VDRE binding, 1,25(OH)_2_D_3_ also regulates the transcription of tumor suppressor p21, which causes cell cycle arrest and initiates apoptosis [14,15].

Previous studies have identified VD target tissues throughout the gastrointestinal tract and in multiple animal species [16]. The injection of radio-labeled 1α,25(OH)_2_D_3_ into mice, rats, hamsters and zebrafish has revealed many sites for ligand binding in the colon, stomach and small intestine [16]. Moreover, VDR expression has been identified in the gastrointestinal tracts of mice, rats and humans [17,18,19]. The roles of VD and VDR in the intestine have been well studied. Adequate calcium absorption in the intestine is maintained by VD targets such as intestinal plasma membrane calcium ATPase (PMCA) and TRPV6 [20].

At the cellular level, different immune cells involved in gastrointestinal immunity express the VDR and they are part of VD production, such as B and T lymphocytes, macrophages and dendritic cells [21]. Within the stomach, the scattered cells in the pyloric antrum and the isthmus region in the corpus were discovered as target tissues of 3H-1α,25(OH)_2_D_3_ [16]. The normal function of VD in the stomach is yet to be explored.

Interestingly, the VDR was found to be poorly expressed or downregulated in malignant gastric tissue [22] and tissues obtained from Barrett’s esophagus [19]. It was found that the VDR was expressed at a higher level in moderately differentiated premalignant gastric tissues than in poorly differentiated premalignant gastric tissues [19,22]. In addition, it was reported that VD deficiency is associated with other gastrointestinal diseases such as inflammatory bowel disease, colorectal cancer and *Helicobacter pylori* infection [23,24,25]. Thus, it is fundamentally important to define the VDR expression patterns and function in normal gastric tissues to provide basic information for the understanding of the pathogenesis of gastric cancer.

The stomach in humans and rodents is structurally subdivided into four parts: the cardia body (or corpus), the fundus (or forestomach) and the pylorus, which includes the antrum and sphincter [26,27]. In rodents, the epithelial layer of the corpus, pylorus and cardia is organized to form tubular glands, which are populated with different epithelial cell types [28]. There are four unique regions in the corpus gastric gland: the pit, isthmus, neck and base. The isthmus region contains stem cells and their immediate descendants (progenitors) that give rise to the cells of the corpus gland upon differentiation. The luminal surface and the pit region contain mucous cells that secrete mucus. The neck region contains mucus-secreting neck cells. Parietal cells produce hydrochloric acid and enteroendocrine cells are dispersed all over the gland regions. The pepsinogen is secreted by the zymogenic (chief) cells, which are located only in the basal region [26,29]. The antral gastric gland is composed of surface mucous cells in the pit region and gland mucous cells in the basal region while the antral stem/progenitor cells in the isthmus/base and enteroendocrine cells are scattered throughout [30,31]. Stem cells not only maintain gastric epithelial homeostasis but also continuously proliferate and differentiate and form all kinds of specialized gastric epithelial cells [28,32]. Although previous studies reported correlations between cancer progression, Ca^2+^ activity and VDR signaling, the clear role of the VDR in the stomach, as well as its role in gastric homeostasis, remains obscure. Therefore, this study was planned to determine the normal expression, distribution and cellular expressions of the VDR in gastric epithelia and the effects of VD3 deficiency on gastric stem cell proliferation and their differentiated descendants. We used immunohistochemistry, real-time PCR and RT-PCR to test our hypothesis that the differentiation and proliferation of gastric epithelial cells are dependent on the VDR function.

## 2. Materials and Methods

### 2.1. Mice and Their Diets

The ethics committee of the United Arab Emirates University approved this study (UAEU, ERA_2017_5684). C57BL/6J mice were kept in autoclaved filter-top cages and handled in a laminar flow hood at the facility allocated for animal research at the College of Medicine and Health Sciences at UAEU.

Three-week-old weaned male mice (*n* = 30) were distributed into three equal groups based on light exposure and diet. The control group was kept on a standard rodent diet, AIN-93G, which included 1000 international units (IU) of VD_3_ (D10012Gi, Research Diet, New Brunswick, NJ, USA) in a 12 h dark/light cycle (SDL). The second group was given the standard diet and maintained in the dark with no overhead light (SDD). The third group (VD_3_-deficient; VDD) was maintained on a growing rodent diet, AIN-93G, with a 25 IU VD_3_/kg diet (D17053003i, Research Diet) with no light source provided. These conditions were kept for three months for all mice.

### 2.2. Histology

Gastric tissues were collected from the euthanized mice and fixed with Bouin’s fixative for 24 h. Ethanol was used to dehydrate the tissue, xylene was used to clear the tissue and paraffin was then used to embed the tissue. Hematoxylin and eosin (H&E) or periodic acid–Schiff (PAS) were used to stain the tissue sections (5 μm). Several tissue sections were processed for immunoperoxidase and immunofluorescent staining. The images were obtained using an Olympus microscope IX83 (Tokyo, Japan).

A PAS staining kit (Newcomer Supply, Middleton, WI, USA) was used to prepare the samples for imaging. The slides were incubated with periodic acid (0.5%) for 10 min and then with the Schiff reagent for 20 min. The tissues were counterstained with the hematoxylin solution for 2 min.

Immunohistochemistry was performed to visualize the VDR and H,K-ATPase β (HK β). Briefly, 1% bovine serum albumin (blocking reagent) was used to incubate the sections for 1 h at room temperature. It was then incubated overnight at 4 °C with an anti-VDR rat polyclonal antibody (ab115495, dilution 1:150, Abcam, Cambridge, UK) and anti-HKβ rabbit polyclonal primary antibody (ab176992, dilution 1:100, Abcam, Cambridge, UK). Goat anti-rat IgG (H + L) antibodies, which were conjugated with Biotin-SP (112-065-003, dilution 1:1000, Jackson Immunoresearch, West Grove, PA, USA), and goat anti-rabbit IgG H&L (TRITC) (ab6718, Abcam) antibodies were then added and incubated at room temperature (22 °C) for 1 h. The phosphate-buffered saline (PBS) was used to wash the sections, which then were incubated with blocking reagent ExtrAvidin^®^-Peroxidase (Thermo Fisher Scientific, Waltham, MA, USA) at a 1:1000 dilution ratio at room temperature. The PBS was then used for washing. The sections were then treated with 3,3′-diaminobenzidine in urea (1:1) for 6 min and counterstained with hematoxylin.

The immunostaining with 5-bromo-2′-deoxyuridine (BrdU) was performed on VDD, SDL and SDD mice (*n* = 5) that had been injected with 120 mg/kg body weight of BrdU (B5002, Sigma Aldrich, St. Louis, MO, USA) intraperitoneally 2 h before euthanasia. The monoclonal mouse antibodies, anti-BrdU (MI-11-3, dilution 1:500, MBL Life Science, Woburn, MA, USA), were then used to incubate the sections at 4 °C for 1 h followed by incubation for 1 h with goat anti-mouse IgG (H + L), biotin-SP-conjugated (115-065-003, Jackson Immunoresearch, dilution 1:500).

Antigen retrieval was performed with co-immunofluorescence staining using a citrate buffer for 20 min at 96 °C. The tissue sections were incubated in 0.1% Triton X-100 in PBS followed by a 1 h blocking reaction with 1% bovine serum albumin and incubation overnight at 4 °C with a rat primary antibody, anti-VDR (ab115495, dilution 1:150, Abcam). The PBS was used to wash the sections and then treated with Alexa Fluor 448 or Cy3-conjugated goat anti-rat secondary antibodies (112-545-003 or 112-165-003, Jackson ImmunoResearch, dilution 1:500) for 1 h at room temperature. The sections were washed with PBS solution and incubated with rabbit anti-HKβ (parietal cell marker) and then with goat anti-rabbit secondary antibodies as described above. Several anti-VDR incubated tissue sections were also incubated with lectins specific for surface mucous cells (*Ulex europaeus* agglutinin I or UEA-1) or mucous neck cells (*Griffonia simplicifolia* II or GSII) that were conjugated with rhodamine or fluorescein, respectively (RL-1062 or L21415, Life Technologies, Carlsbad, CA, USA).

4′,6-diamidino-2-phenylindole (ab104139, Abcam) was used as a mounting medium and as nuclear counterstaining section sample preparations. Negative controls for each experiment excluded primary antibodies.

### 2.3. RNA Separation and cDNA Synthesis

An RNeasy Mini Kit (Qiagen, Hilden, Germany) was used to collect total RNA from different parts of the stomachs of the mice (forestomach, corpus and antrum) using the manufacturer’s directions. RNA purification was performed using an RNase Free DNase kit (Qiagen). The cDNA was synthesized by reverse transcribing 1 μg RNA using the iScript™ cDNA synthesis kit (Bio-Rad, Hercules, CA, USA) following the manufacturer’s protocol.

### 2.4. Reverse Transcription PCR (RT-PCR)

The expression patterns of *Cyp2r1, Vdr*, *Cyp27b1*, *Cyp24a1 and Cyp27a1* in the gastric tissue of normal mice were analyzed. A GoTaq Flexi DNA polymerase kit (Promega, Madison, WI, USA) was used to conduct the RT-PCR with cDNA using the manufacturer’s instructions and the primers are shown in Table S1. Gel electrophoresis was used to separate PCR products with ethidium bromide in 2% agarose and visualized with a Gel Doc™ EZ Imager (Bio-Rad).

### 2.5. Quantitative Real-Time PCR (qRT-PCR)

Quantitative real-time PCR (qPCR) using a QuantiStudio^®^ 5 Real-Time PCR instrument (Applied Biosystems, Foster City, CA, USA) was used for the differential expression of genes specific for gastric epithelial cell lineages, cell proliferation or direct target VDR genes. Green dye SYBR was employed for the quantitation of double-stranded DNA after an individual cycle. The Supplementary Data Table S1 lists the primers used. Reactions with 40 amplification cycles were performed with each cycle including denaturation for 15 s at 95 °C, annealing for 1 min at 60 °C and extension for 15 s at 95 °C. The melt curve stage was performed at 60 °C followed by dissociation for 1 s at 95 °C. The samples were tested in triplicate and the expressions of gene levels were measured using the comparative cycle threshold method (ΔΔCT method). The expression was then normalized to *Gapdh*.

### 2.6. Mass Spectrometric Measurement of Serum Vitamin D

Blood samples were obtained immediately by a cardiac puncture technique in anaesthetized animals. Approximately 1 mL of blood was collected into BD vacutainers (Ref. 367957; BD Biosciences, Franklin Lakes, NJ, USA). Centrifugation was used at 906× *g* (3500 rpm) for 15 min at 4 °C to separate the serum and then stored at −80 °C. The analysis of serum 25(OH)D required the calculation of the total concentration of 25(OH)D, which is the sum of [25(OH)D3 + 25(OH)D2]. Liquid chromatography coupled to tandem mass spectrometry (LC-MS/MS) was performed to quantify 25(OH)D3 and 25(OH)D2. This was followed by adding an internal standard 25(OH)D3(6,9,9-D3) to each sample and vortex mixing for 1 min. One milliliter of hexane in ethyl acetate (9:1; *v*/*v*) was then added to each sample for liquid-liquid extraction (LLE). This sample was centrifuged at 3000× *g* for 30 min and the top organic layer was stored whereas the lower layer was further extracted by adding another round of hexane and ethyl acetate (9:1; *v*/*v*) and centrifuging two more times. The extracts from all three samples were pooled together and dried using N2 gas at room temperature in a sample concentrator (Stuart sample concentrator model no SBHCONC/1, Stuart Equipment’s, Staffordshire, UK). Calibrants containing the spiked samples at a known concentration were used to prepare the calibration curve. Quality controls composed of spiked samples prepared at a known concentration at three different levels were processed using the same LLE along with the samples. Finally, the dried residue was resuspended using a mixture of methanol/water (75:25, *v*/*v*). The samples were analyzed using the Ultra High Pressure Liquid Chromatography (UHPLC) system coupled to a tandem mass spectrometer, model 8060 system (Shimadzu, Kyoto, Japan).

### 2.7. Measurement of Gastric Acid

Stomachs harvested from SDL, SDD and VDD mice were cut up along the greater curvature. A total of 1 mL of 0.9% NaCl (pH 7.0) was used to rinse the stomachs and centrifuged at 1848× *g* for 10 min. This was followed by the collection of the supernatant. The H^+^ concentration was quantified by performing manual titrations that compared the supernatant concentration with the 0.005 M NaOH. Finally, these results were then normalized to the weights of their bodies.

### 2.8. Statistical Analysis

GraphPad Prism 7.0.3 (GraphPad, Inc., San Diego, CA, USA) was used for the statistical analysis. A one-way analysis of variance (ANOVA), Student’s *t*-tests or Dunnett’s multiple comparisons post-test were used for the data analysis as required. Data were presented as the means ± standard deviation (SD) and the results were considered significant if *p*-values < 0.05.

Fiji-ImageJ software (version 1.52n, Tokyo, Japan) was used for the analysis to enumerate BrdU-labeled cells and parietal cells as well as to measure the HK β pixel intensity.

## 3. Results

### 3.1. Expression of the VDR in Normal Gastric Epithelial Tissues

**Figure 1:**
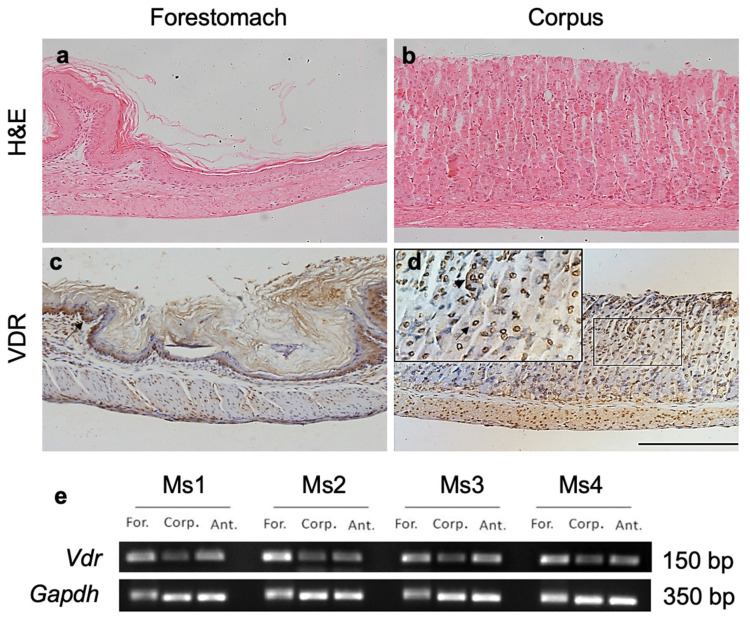
VDR expression in murine stomachs. H&E staining of the (**a**) forestomach and (**b**) corpus and the immunoperoxidase analysis of adult normal mouse stomachs (2–4 months old, *n* = 4) using a rat polyclonal anti-VDR antibody showing positive signals in both the (**c**) forestomach and (**d**) corpus. VDR expression was observed primarily in the nucleus ((**c**), arrow) with an occasional cytoplasmic expression ((**d**), arrowhead). Scale bar, 200 μm. (**e**) Qualitative PCR analysis of *Vdr* expression in four adult normal mice (Ms1–4) produced bands in the forestomach (For.), corpus (Corp.) and antrum (Ant.) with the most intense bands observed in the forestomach. *Gapdh* was used as an internal control, full figure please see Supplementary Materials File 1.

The distribution of the VDR in normal mouse stomachs was determined; VDR-specific antibodies were used for immunolocalization (Figure 1). We observed the binding of VDR antibodies along the forestomach and epithelial lining of the corpus (Figure 1c,d). Cells in all of the parts of the gastric gland of the corpus including the pit, isthmus, neck and base expressed the VDR. VDR localization was primarily nuclear with occasional cytoplasmic expression (Figure 1d insert, arrow and arrowhead). PCR detection of *Vdr* expression confirmed that *Vdr* was expressed in the forestomach and corpus regions as well as the pyloric antrum of the stomach (Figure 1e).

### 3.2. The VDR Is Expressed by Gastric Parietal and Mucous Cells

**Figure 2:**
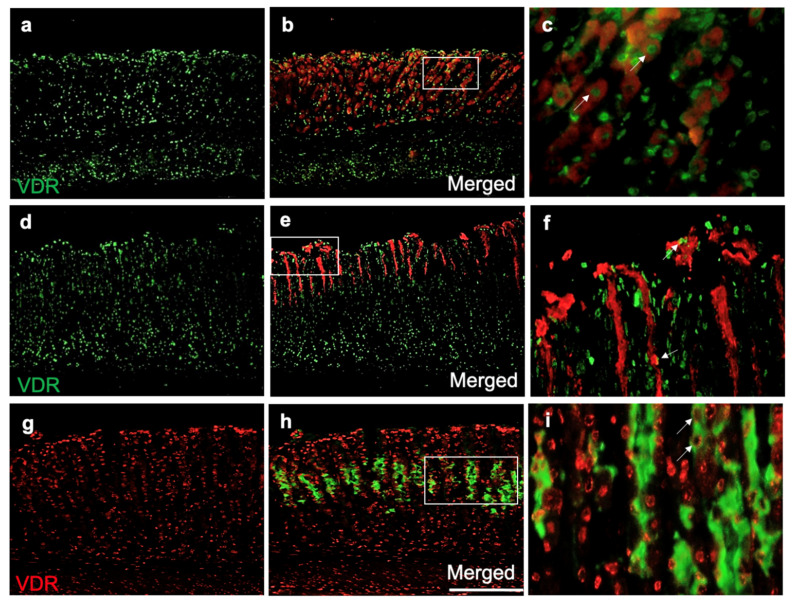
Expression of the VDR in the gastric epithelial cells of 2–4-month-old mice. (**a**,**d**,**g**) Anti-VDR antibody colocalized with (**b**) a parietal cell-specific HK β antibody, (**e**) surface mucous cell-specific UEA-1 lectin and (**h**) mucous neck cell GSII lectin. Scale bar, 200 μm. VDR expression in (**c**) parietal cells, (**f**) surface mucous cells and (**i**) mucous neck cells. Scale bar, 100 μm. *n* = 3.

To determine which gastric epithelial cells express the VDR and therefore respond to VD_3_, immunofluorescent staining was performed on tissue sections from normal mice. The VDR antibody was colocalized with the parietal cell-specific marker, HK β (Figure 2a–c). The colocalization of the VDR was also observed with UEA-1-labeled (Figure 2d–f) and GSII-labeled (Figure 2g–i) surface mucous cells and mucous neck cells, respectively.

### 3.3. Gastric Expression of CYP Enzymes Involved in Regulating VD_3_

**Figure 3:**
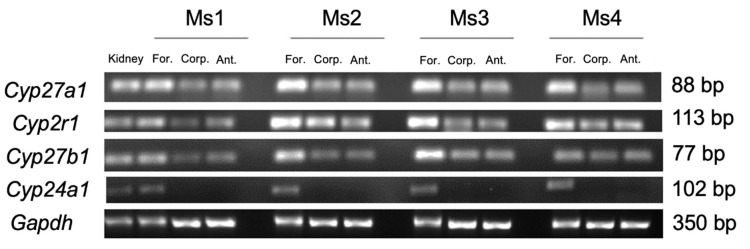
CYP enzyme expression in the stomachs of four 2–4-month-old normal mice (*n* = 4). *Cyp2r1*, *Cyp27a1* and *Cyp27b1* were expressed in the forestomach (For.), corpus (Corp.) and antrum (Ant.) with the highest expression observed in the forestomach. *Cyp24a1*, which is responsible for VD degradation, was expressed only in the forestomach. Kidney RNA and *Gapdh* were used as positive controls and internal controls, respectively.

Cytochrome P450 enzymes regulate the expression of serum VD_3_. These enzymes are expressed in the kidney and liver and are involved in both VD_3_ activation (CYP2R1, CYP27A1 and CYP27B1) and degradation (CYP24A1). To determine if these enzymes are expressed in the stomach, a PCR analysis was performed (Figure 3). *Cyp2r1*, *Cyp27a1* and *Cyp27b1* were expressed in all regions of the stomach with the highest levels in the forestomach. In contrast, the expression of *Cyp24a1* was detected only in the forestomach (Figure 3).

### 3.4. Establishment of a VDD Mouse Model

To understand the nutritional role of VD in gastric cell homeostasis, a specific diet deficient in VD was provided to mice. This very low VD-supplemented diet was selected to avoid negative feedback consequences such as high serum calcium and phosphate. All mice tolerated the diets well. The serum levels of total 25(OH)D and [25(OH)D3 + 25 (OH)D_2_] were quantified. The Holick classification for VD deficiency in humans was followed [33]; specifically, VD deficiency was considered to be 25(OH)D < 20 ng/mL, insufficiency of 25(OH)D was defined as a concentration between 21–29 ng/mL and an optimal level of 25(OH)D was considered to be 25(OH)D > 30 ng/mL.

**Figure 4:**
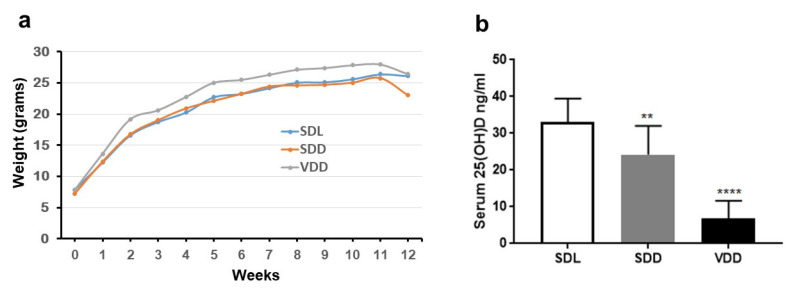
Effect of a VD-deficient diet on weight and VD serum levels. Diets containing 25 IU/kg-VD3 (VD3, deficient diet) or 1000 IU/kg (standard diet) were administered to approximately 4-month-old mice (*n* = 7/group) to assess their effects on serum 25(OH)D and weight. (**a**) VDD mice gained more weight than the controls. (**b**) VDD mice exhibited a significant reduction in 25(OH)D serum concentration presented as the mean (ng/mL) ± SD. A one-way ANOVA was used for the serum 25(OH)D analysis and a two-way ANOVA was performed for the weight analysis. ** *p* < 0.01, **** *p* < 0.0001.

Body weights were assessed for 3 months; there was a slight increase observed in the VDD group when compared with the control mice. This finding was not in agreement with a previous study that reported VD deficiency was associated with obesity [34]; however, there was no significant difference between this and the control group (*p*-value > 0.05, Figure 4a). Serum 25(OH)D was reduced from 33.14 ng/mL in the control group to 6.81 ng/mL in the VDD group due to the lower VD_3_ dietary intake for 3 months. Interestingly, our results showed that VD insufficiency (24.18 ng/mL) could be produced in mice by removing the overhead light only (normal diet) to prevent the production of endogenous VD (Figure 4b). We also found that none of the mice in any of the groups expressed any signs of hair loss or reduced physical activity and all mice behaved normally.

### 3.5. Low Gastric Acid Content along with Increased Gastrin Gene Expression in the VDD Stomach

**Figure 5:**
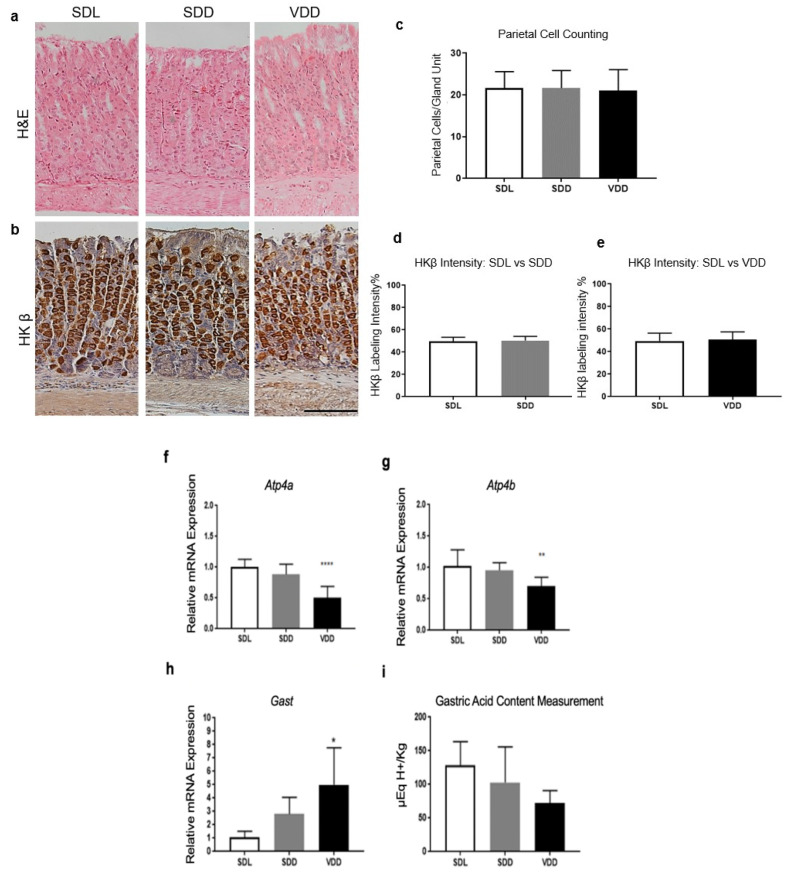
Gastric acid content is reduced in VD-deficient mouse stomachs. (**a**) H&E staining of the gastric mucosae of SDL, SDD and VDD mice. (**b**) Immunohistochemical detection of parietal cell-specific HK β. No change was observed in the (**c**) number of parietal cells or intensities of HK β antibody staining in either the (**d**) SDD and (**e**) VDD groups compared with the SDL control group. Scale bar, 200 μm. A significant decrease in (**f)** *Atp4a* and (**g**) *Atp4b* expression was observed in the VDD group compared with the SDL controls. (**h**) *Gast* expression increased while (**i**) gastric acid content decreased slightly in VD-deficient mice compared with those in the control mice. All mice were approximately 4 months old (*n* = 7/group). A one-way ANOVA and Student’s *t*-tests were used for the analysis of the data. * *p* < 0.05, ** *p* < 0.01, **** *p* < 0.0001.

After establishing that the VDR was expressed at high levels in the normal stomach tissue, we examined the effects of the deficiency of VD on the gastric target cells of VD, as shown in Figure 5. A microscopic examination of the H&E stained sections of the gastric mucosa revealed no apparent differences in mucosal tissue thickness or overall tissue organization (Figure 5a) except that the parietal cells appeared slightly smaller in the VDD mice than in the controls (Figure 5b). However, the parietal cells exhibited similar HK β staining in all groups (Figure 5d,e). Alternatively, the mRNA expression of *Atp4a* and *Atp4b* was significantly downregulated (*p* = 0.0001 and 0.0083, respectively) in VDD mice compared with control mice (Figure 5f,g). Although we did not see any statistically significant difference, the gastric contents of H^+^ in the SDL and SDD groups were greater than the VDD group, as shown in Figure 5i.

It is established that gastrin regulates the secretion of acid by parietal cells [35]; therefore, the mRNA expression of gastrin was also quantified. Unexpectedly, the relative expression of *Gast* mRNA was significantly upregulated four-fold (*p* = 0.0174) in the VDD group relative to the control group. However, the SDD group did not exhibit a significant upregulation of *Gast* expression in the antral tissues (Figure 5h).

### 3.6. A Noticeable Increase in Gastric Stem/Progenitor Cell Proliferation in the VDD Group of Mice

**Figure 6:**
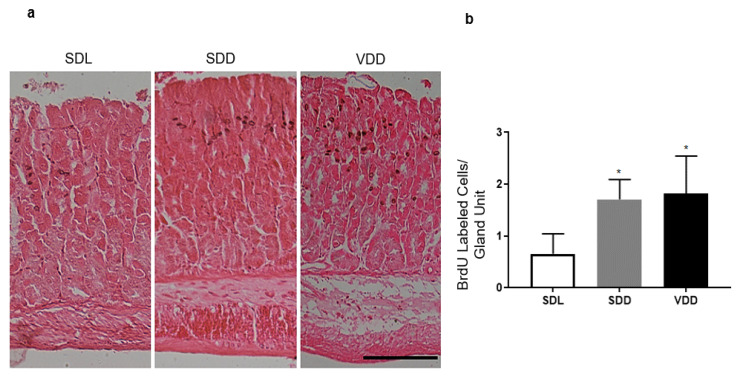
VD deficiency is associated with an increased proliferation. (**a**) Immunohistochemical analysis of BrdU-labeled proliferating cells in the isthmus domain of the corpus gastric gland (*n* = 4/group). Scale bar, 200 μm. (**b**) Increased BrdU-label in the SDD and VDD groups compared with the SDL group. The data analysis was performed using a one-way ANOVA, * *p* < 0.05.

BrdU immunolabeling was performed to test whether a high gastrin expression in the stomachs of VDD mice was associated with a change in stem cell proliferation in the gastric mucosa. Proliferating cells were quantified by calculating an average number of positive BrdU cells in the corresponding locations of the gastric mucosal histological sections. This demonstrated an increase in the stem cell proliferation in the isthmus region of the corpus gastric gland in VDD and SDD mice compared with the controls (*p* = 0.029 and 0.025, respectively; see Figure 6).

### 3.7. Gastric Differentiation Markers and Vdr Expression Are Affected by Vitamin D Status

**Figure 7:**
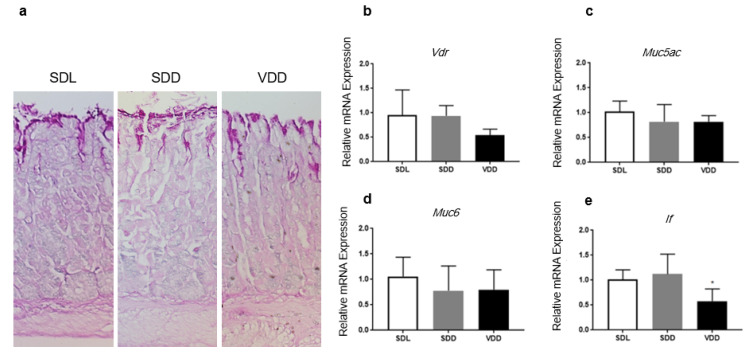
PAS staining and expression of cellular differentiation markers in VD-deficient mice. (**a**) No apparent difference in the PAS staining of gastric mucosa. RNA was obtained from the corpus regions of SDD, SDL and VDD mice (*n* = 7/group) to evaluate the expression of representative genes by qPCR. Relative mRNA expression of (**b**) *Vdr*, (**c**) *Muc5ac*, (**d**) *Muc6* and (**e**) *If*. If only showed a significant decrease in VD-deficient mice when compared with the controls. The data analysis was performed using a one-way ANOVA, * *p* < 0.05.

No change was observed in the PAS staining carried out on the surface mucous cells in the gastric tissue samples between the three treatment groups (Figure 7a). To examine the regulatory effect of VD_3_ on the *Vdr* transcription and gastric epithelial cell lineage-specific genes and targeted genes of 1α,25(OH)_2_D_3_ (*p21, Pthlh* and *Trpv6*), RNA extracted from the corpus region was used to carry out qPCR. In mice with VD_3_-deficient diets, the expression of *Vdr* mRNA decreased (*p* = 0.05) compared with the control mice group (Figure 7b). We then investigated the mucous cells in the gastric epithelia in mice with low and normal levels of serum VD by analyzing and comparing the relative expression of *Muc6* and *Muc5ac* markers for mucous neck cells and surface mucous cell, respectively. We found a small but not statistically significant reduction in the transcription levels of the two important genes in VDD and SDD mice compared with SDL mice (Figure 7c,d). However, the expression of *If*, which is specific for chief cells, was significantly downregulated (*p* = 0.0309) in the VDD group when compared with the control (Figure 7e).

### 3.8. The Expression of the Target Genes of 1α,25(OH)_2_D_3_ in Response to Vitamin D Status

**Figure 8:**
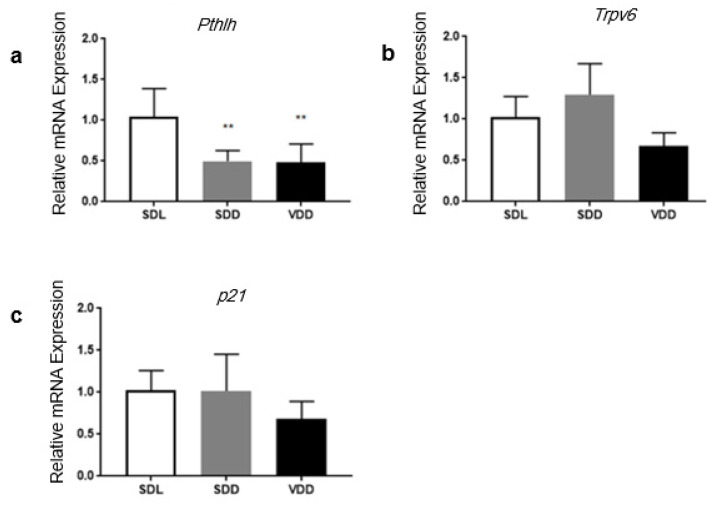
The expression of 1α,25(OH)_2_D_3_ targeted genes. RNA was extracted from the corpora of approximately 4-month-old SDL, SDD and VDD mice (*n* = 7/group) for the qPCR analysis. Relative mRNA expression of (**a**) *Pthlh*, (**b**) *Trpv6* and (**c**) *p21* all showed a decline in VD-deficient mice compared with the SDL controls. A one-way ANOVA was employed for the data analysis. ** *p* < 0.01.

The transcription of several genes is regulated by 1α,25(OH)_2_D_3_ through the binding of VDRE to the promoters of target genes such as *Trpv6*, *Pthlh* and *p21.* Previously, these genes were reported to be expressed in the stomach [8,10,14]. We found that within the gastric mucosa of VDD mice, the relative mRNA expression of these genes was moderately downregulated when compared with the SDL control mice group (Figure 8a–c). Lastly, it was only possible to detect a statistically significant downregulation for *Pthlh* expression.

## 4. Discussion

A considerable number of studies have reported the physiological functions of the VDR and VD_3_ in rodents and humans. The results of these studies have indicated roles for VD_3_ in calcium homeostasis [36], blood cell differentiation [37] and insulin secretion [38].

The VDR is widely dispersed in multiple organs and tissues in the body as well as in the gastrointestinal tract, specifically in the colon, small intestine and gastroesophageal junction [21,23]. In the stomach tissue, many targets of 1α,25(OH)_2_D_3_ have been detected [16]. In the current study, we demonstrated that the VDR is expressed in different parts of the gastric epithelial tissues. Although Stumpf et al., using autoradiography, found that only chromaffin cells and mucous neck cells of the gastric glands are targets for 1α,25(OH)_2_D_3_ [16], our immunohistochemical analysis revealed a wider cellular scattering of the VDR along the corpus gastric glands including mucous neck cells, surface mucous cells and parietal cells.

We also observed that the expression of both *Vdr* and *Cyp* genes is involved in the breakdown and synthesis of 1α,25(OH)_2_D_3_. These genes were expressed in separate parts of the stomach with the antrum and corpus gastric glands explicitly expressing *Cyp27a1, Cyp2r1* and *Cyp27b1*, which encode the enzymes responsible for producing 1α,25(OH)_2_D_3_ [5,39,40], suggesting that these two regions of the gastric epithelia can locally generate biologically active VD_3_. It has also been reported that *Cyp24a1*, which encodes a protein that is responsible for inactivating 1α,25(OH)_2_D_3_ [41], is regulated in a negative feedback loop by 1α,25(OH)_2_D_3_ through the attachment of VDRE to the promoter region of *Cyp24a1* [42]. Our study demonstrated that the *Cyp24a1* expression is exclusively evident in the forestomach (Figure 3), suggesting an important role of the forestomach in the degradation of 1α,25(OH)_2_D_3_ and the monitoring of VD_3_ local levels.

We investigated the impact of VD deficiency on gastric acidity as well as gastric epithelial cell proliferation and differentiation. It is well known that, in parietal cells, HK-ATPase is responsible for the secretion of acid by exchanging H^+^ for K^+^. Moreover, the gastrin hormone regulates the acid secretion of parietal cells [43]. Herein, we report a reduction in gastric acidity, a decrease in mRNA expression of *Atp4b* and *Atp4a* and an increase in the expression of *Gast* mRNA in VD-deficient mice. Interestingly, Antico et al. found a correlation between low 25(OH)D_3_ serum concentrations and the development of autoimmune-gastritis (AIG) in an Italian study population [44]. They further showed that the deficiency of vitamin D is a risk factor for AIG [44], which results from the production of autoantibodies produced against HK and decreased acid secretion and causes inflammation in gastric mucosa. The advanced stage of the disease finally causes mucosal atrophy [45]. Our data are consistent with those reported by Antico et al., which showed that low 25(OH)D_3_ levels may be a predisposing factor for decreased gastric acid secretion and AIG development.

We also observed that VD-deficient mice showed a significant increase in the proliferation of gastric stem cells (Figure 6). An increased cell proliferation in parathyroid glands has also been reported in rats administered a VD-deficient diet for 3 weeks [46]. Additionally, the colons of *Vdr* knockout mice revealed a significant upregulation in the expression of proliferating cell nuclear antigen (*PCNA*) when compared with the wild type mice [47]. Collectively, these results indicate the significance of the VDR and vitamin D in regulating cell proliferation. Furthermore, higher levels of proliferation in gastric stem/progenitor cells within VD-deficient mice correlates with increased gastrin expression levels as gastrin is a trophic hormone that stimulates proliferation [48,49].

We examined the changes in the relative mRNA expression of *Muc5ac*, *Muc6* and *If*, which are specific for surface mucous, mucous neck and chief cells, respectively. As the serum concentrations of 25(OH)D_3_ were changed in the different study groups, finding a mild downregulation in the expression of all of these differentiation markers was expected. However, we were only able to detect a statistical significance for *If*.

The VDR has been reported to regulate the transcription of the VD_3_ target genes by the binding of VDRE to their promoters [8]. *Trpv6*, a calcium channel, is expressed in the stomach and plays an important role in Ca^2+^ homeostasis in the GI tract [10,11]. *P21* and *Pthlh* are also 1α,25(OH)_2_D_3_ target genes that are expressed in the stomach. PTHLH is regulated by gastrin and it is a growth factor regulator [50] while p21 is a tumor suppressor that causes cell cycle arrest [14]. We found a statistically significant decrease in *Pthlh* in the VD-deficient mice when compared with the control mice (Figure 8). The true role of *Pthlh* in the stomach is yet to be discovered.

## 5. Conclusions

This study revealed and highlighted the importance of VD in a normal stomach. The gastric epithelial parietal and both types of mucus-secreting cells were identified as responsive to VD. Moreover, mice on a VD-deficient diet for 3 months showed lower gastric acid contents and a higher gastrin hormone expression as well as increased cellular proliferation rates. These reported results suggest that the VDR and VD play a significant role in maintaining gastric epithelial stem cell proliferation and differentiation. This work improves our understanding of the relationship between VD deficiency and gastric diseases such as cancer. More research will be required to characterize the behavior of similar genes and proteins in humans with VD deficiency during the multistep process of gastric carcinogenesis.

## Data Availability

The raw data used to generate the graphs are available upon request.

## Supplementary Materials

The following are available online at https://www.mdpi.com/article/10.3390/biology10080705/s1, Table S1: Oligonucleotide primers for the amplification of *Vdr* and *Cyp.* File 1: Original figures.
