# Supplementary material for: Vitamin D Is Necessary for Murine Gastric Epithelial Homeostasis
Source: Biology (Basel). 2021 Jul 23;10(8):705. doi: 10.3390/biology10080705 (PMC8389223; doi:10.3390/biology10080705)
Supplement: Supplementary file 1 [file biology-10-00705-s001.zip › biology-1237266 -Supplementary Materials -main.pdf]

Table S1: Oligonucleotide primers for the amplification of *Vdr* and *Cyp*

	Gene	Forward	Reverse	Product size (bp)
**Receptor**				
1	*Vdr*	5'- GAATGTGCCTCGGATCTGTGG-3'	5'- ATGCGGCAATCTCCATTGAAG-3'	150
**Vitamin D metabolites enzymes (*CYP* genes)**				
2	*Cyp27b1*	5'- TCAGCAGGCATCGCAGAAC-3'	5' GCATTGGATCCTGAGGAATGA-3'	77
3	*Cyp24a1*	5'- CTCCCTATGGATGCAGTATGTATAGTG-3'	5'- TTTAAAAACGTTGTCAGTAGGTCATAACT-3'	102
4	*Cyp2r1*	5'- CAGAAAGACGCTGAAAGTGCAA -3'	5'- CAGTGTATTTGTGTTTACTTGGCTTTATAA-3'	113
5	*Cyp27a1*	5'- GGAGGGCAAGTACCCAATAAGA -3'	5'- TGCGATGAAGATCCCATAGGT-3'	88
**Differentiation markers**				
6	*Atp4a*	5'-TGTACACATGAGAGTCCCCTT G-3'	5'-GAGTCTTCTCGTTTTCCACACC-3'	157
7	*Atp4b*	5'-AAC AGA ATT GTC AAG TTC CTC-3'	5'-AGA CTG AAG GTG CCA TTG-3'	140
8	*Gast*	5'-GGACCAGGGACCAATGAGG-3'	5′-CCAAAGTCCATCCATCCGTAGG-3'	173
9	*If*	5'-CTT GGC CCT GAC CTG TAT GT-3'	5'-TAG GTT GCT CAG GTG TCA CG-3'	191
10	*Muc5ac*	5'-GTGGTTTGACACTGACTTCCC-3'	5'-CTCCTCTCGGTGACAGAGTCT-3'	103
11	*Muc6*	5'-AGCCCACATTCCCTATCAGC-3'	5'-CACAGTGGAAGATTGCGAGAG-3'	192
**VDR target genes**				
12	*Pthlh*	5- GACGTACAAAGAACAGCCACTCA-3	5'- TTTTTCTCCTGTTCTCTGCGTTT-3'	81
13	*Trpv6*	5-TGACCCCTAAGGATGACCTCC-3	5'-CTGTCCAAAGAATCGAGTGACC-3'	122
14	*p21*	5-CGCTGTCTTGCACTCTGGT-3	5'-CGTTTTCGGCCCTGAGATGTT-3'	76
**Housekeeping gene**				
15	*Gapdh*	5'-TCAAGAAGGTGGTGAAGCAGG-3'	5'-TATTATGGGGGTCTGGGATGG-3'	350
