# Supplementary material for: Vitamin D Is Necessary for Murine Gastric Epithelial Homeostasis
Source: Biology (Basel). 2021 Jul 23;10(8):705. doi: 10.3390/biology10080705 (PMC8389223; doi:10.3390/biology10080705)

Figure 1-e:

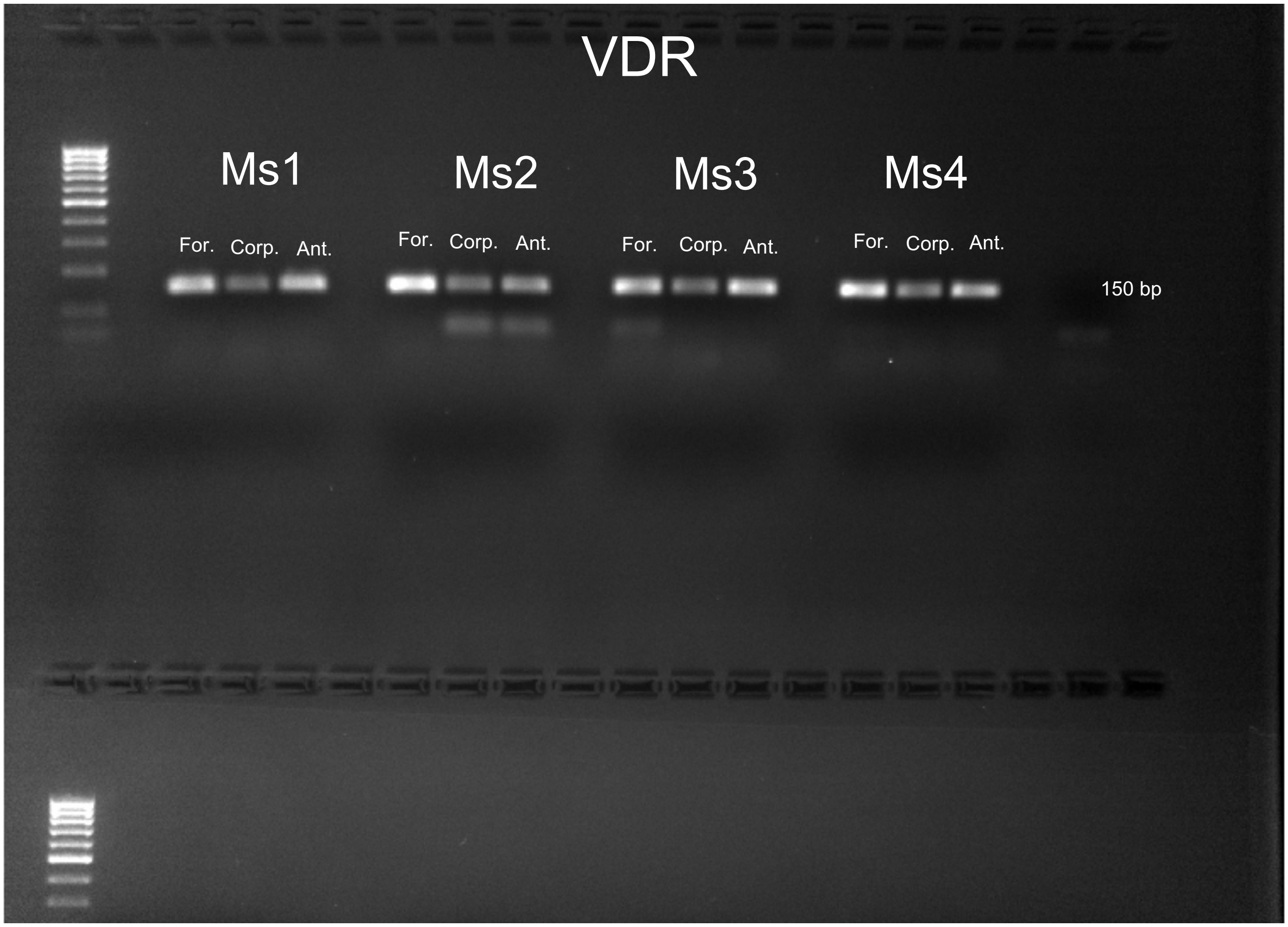

- Ladder used: 1000 , min is 50 bp

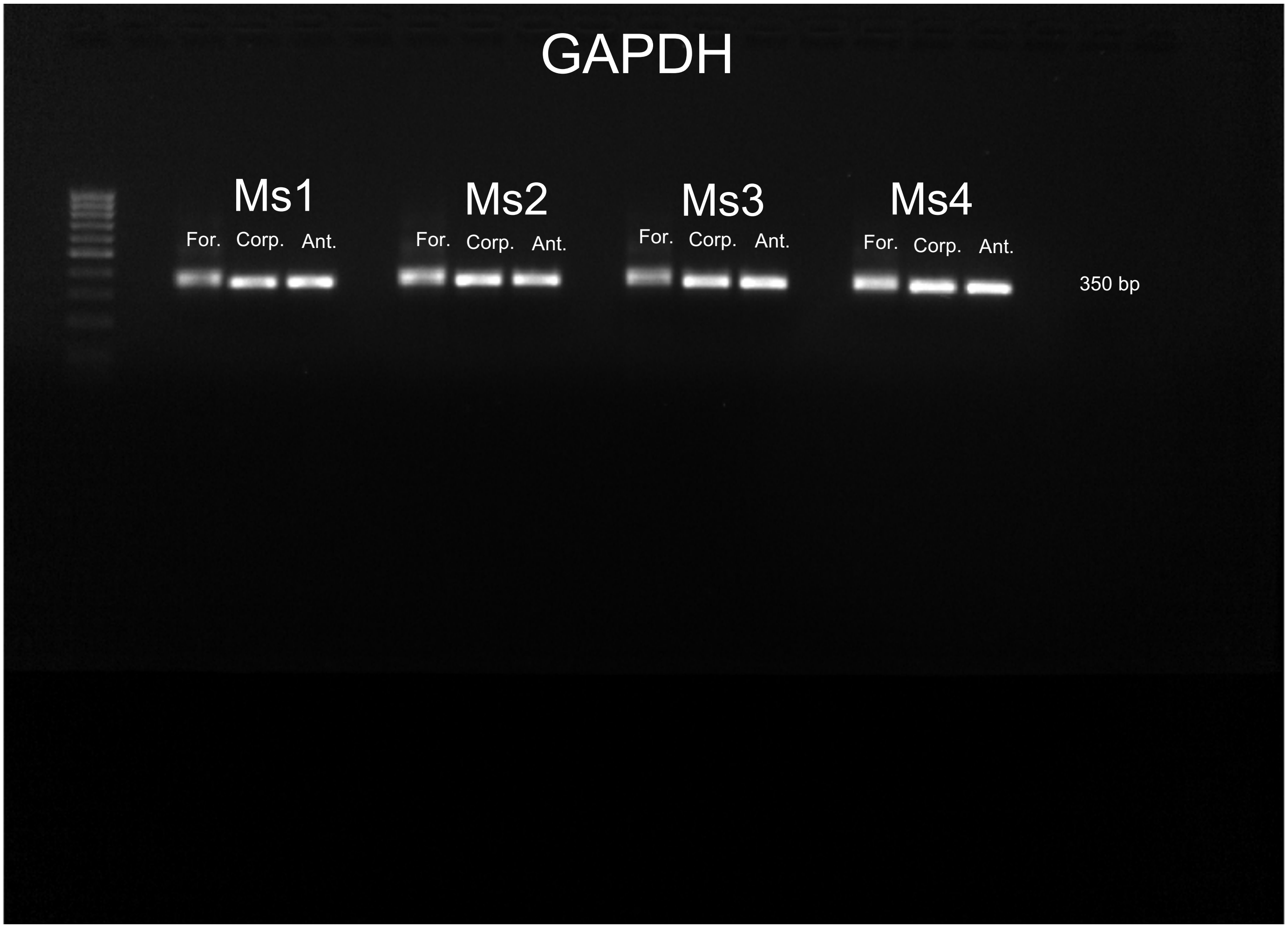

- Ladder used: 1000 , min is 50 bp

Figure 3:

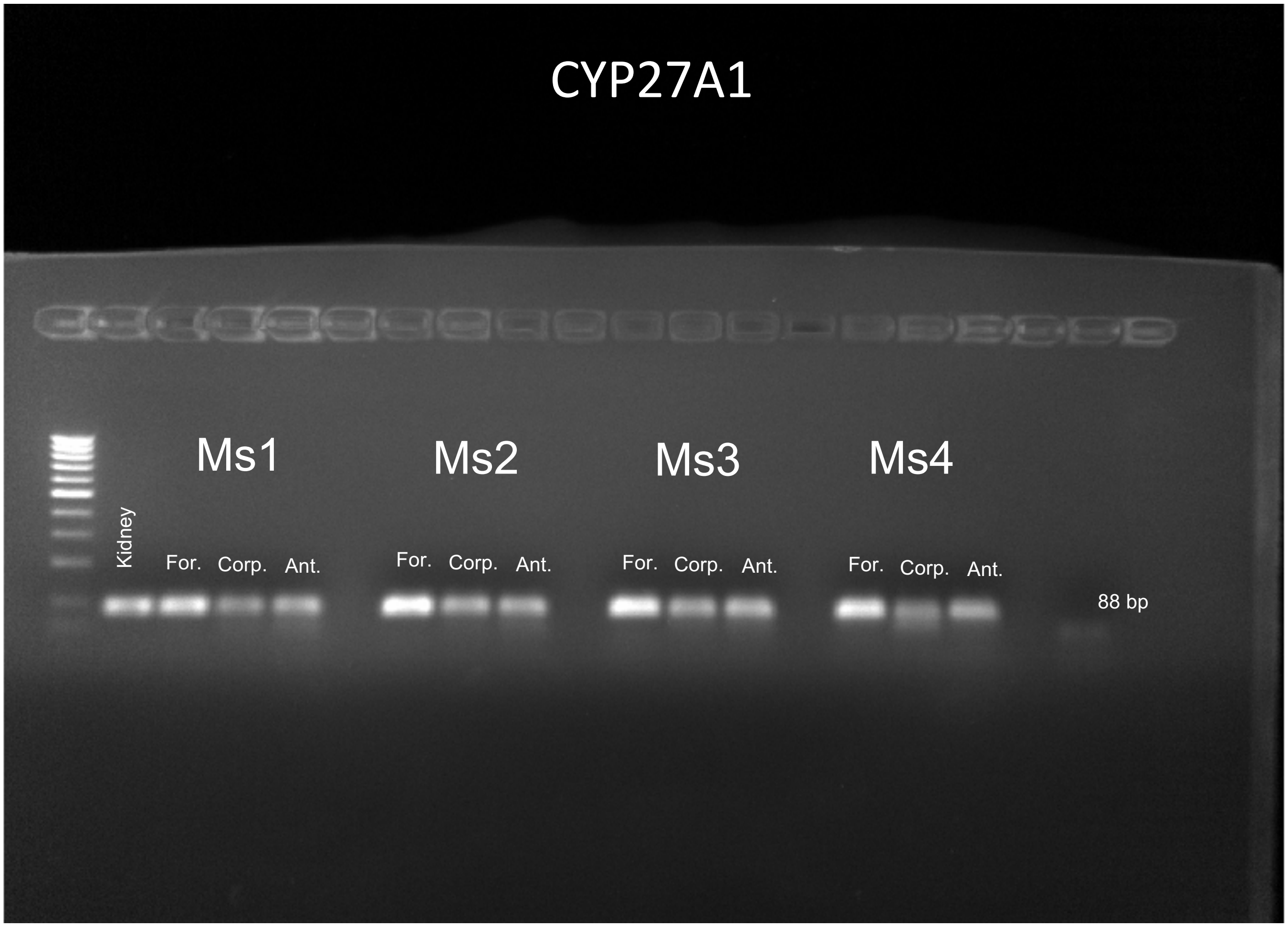

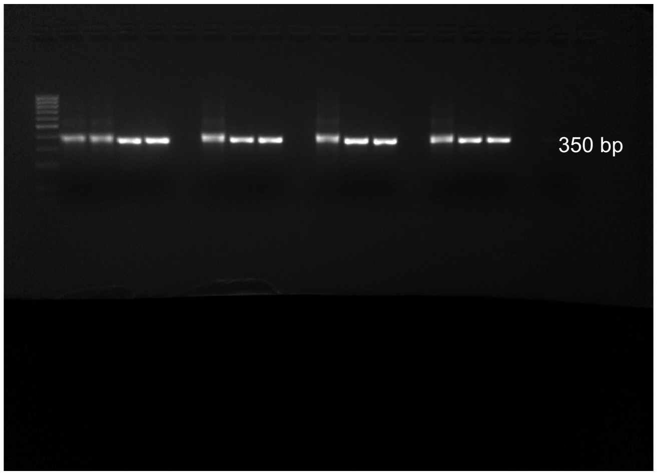

- Ladder used: 1000 , min is 50 bp

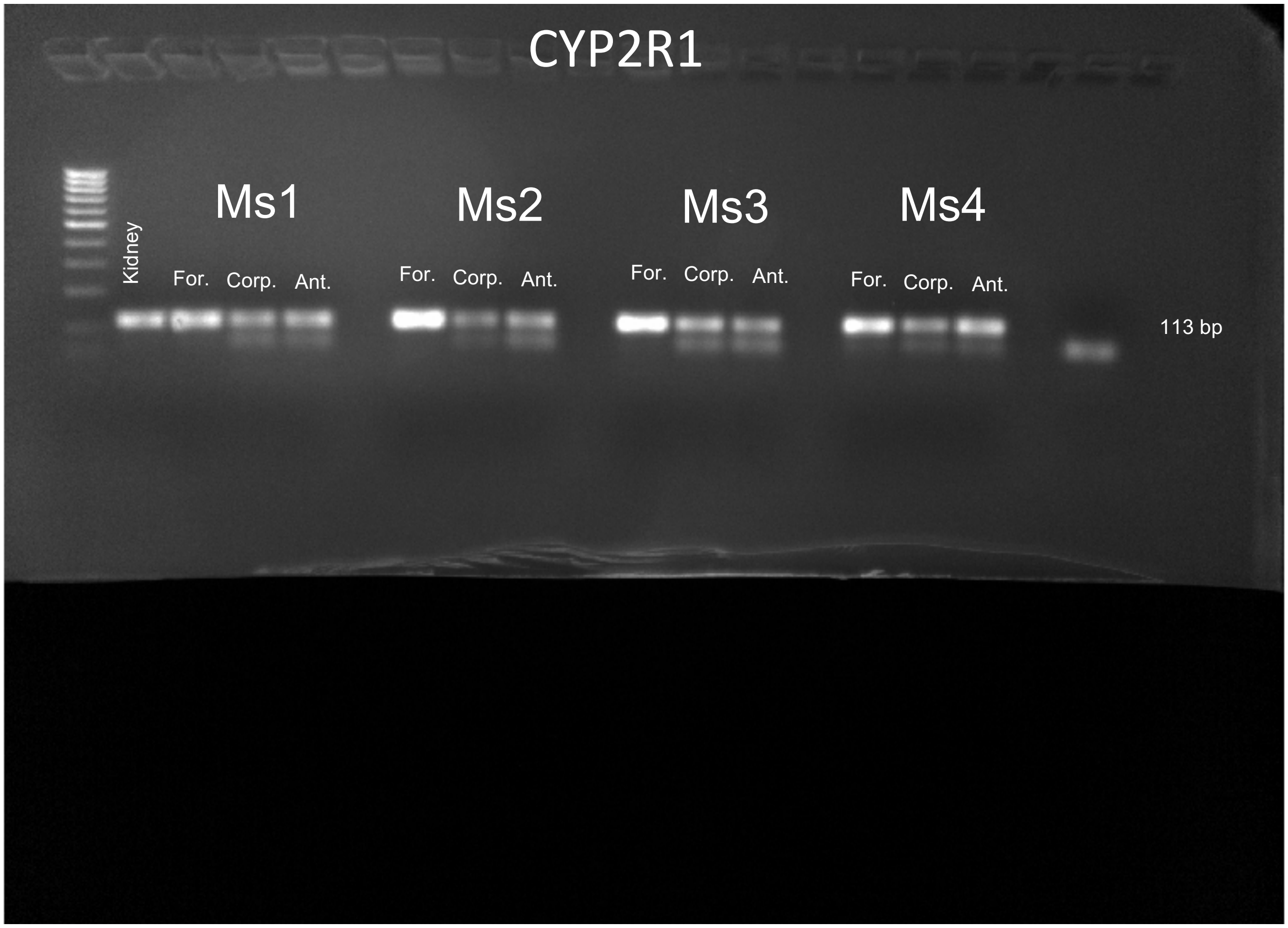

- Ladder used: 1000 , min is 50 bp

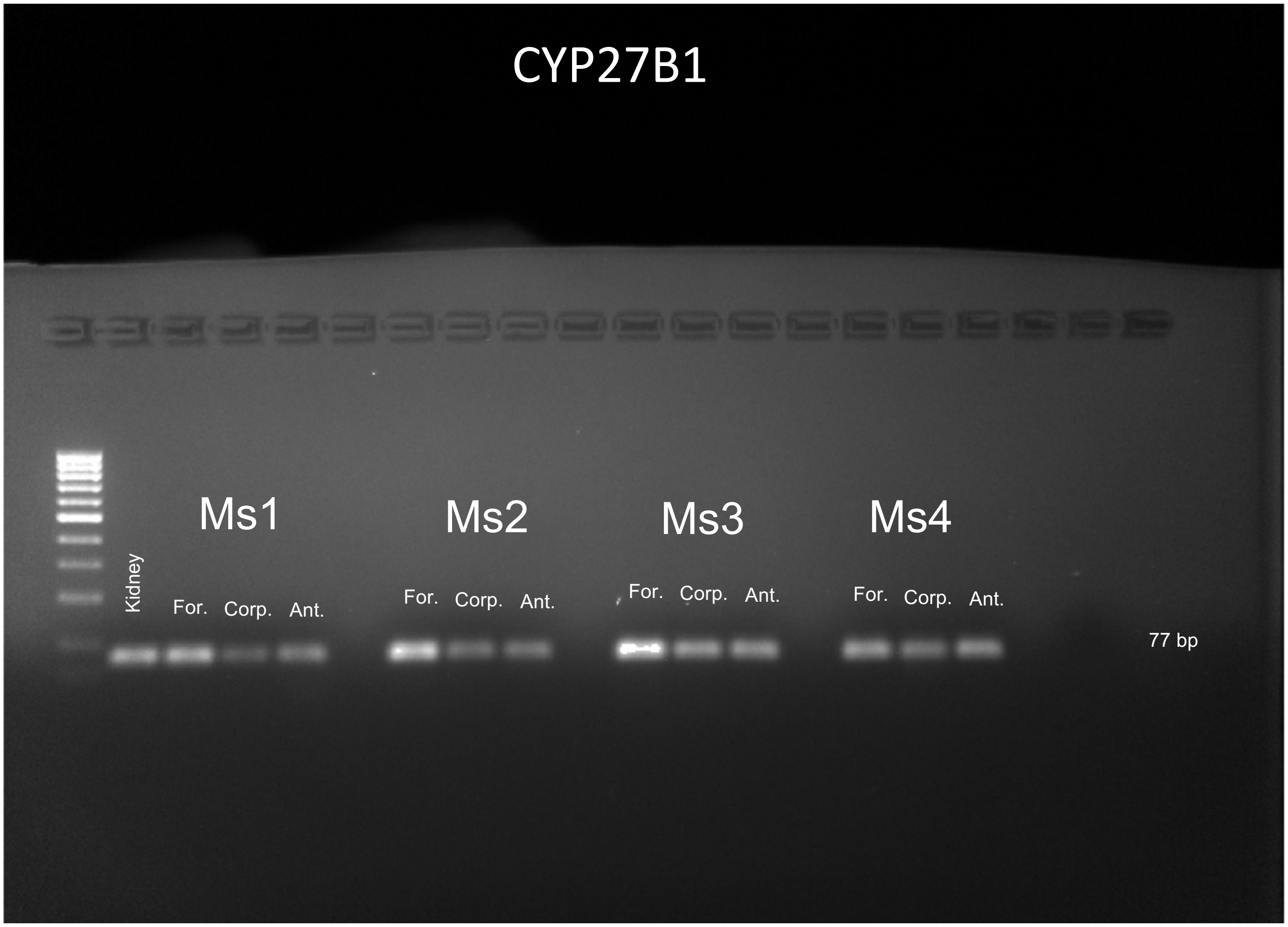
CYP27B1
Ms1
Ms2
Ms3
Ms4
Kidney
For.
Corp.
Ant.
For.
Corp.
Ant.
For.
Corp.
Ant.
For.
Corp.
Ant.
77 bp

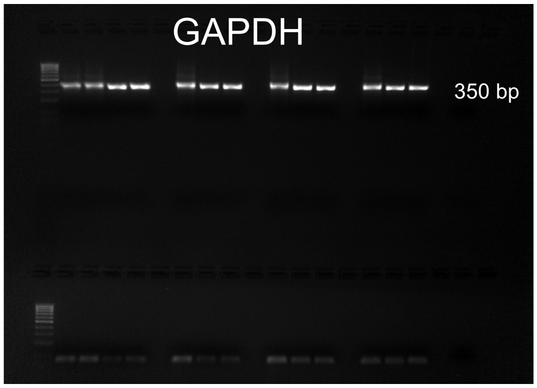
GAPDH
350 bp

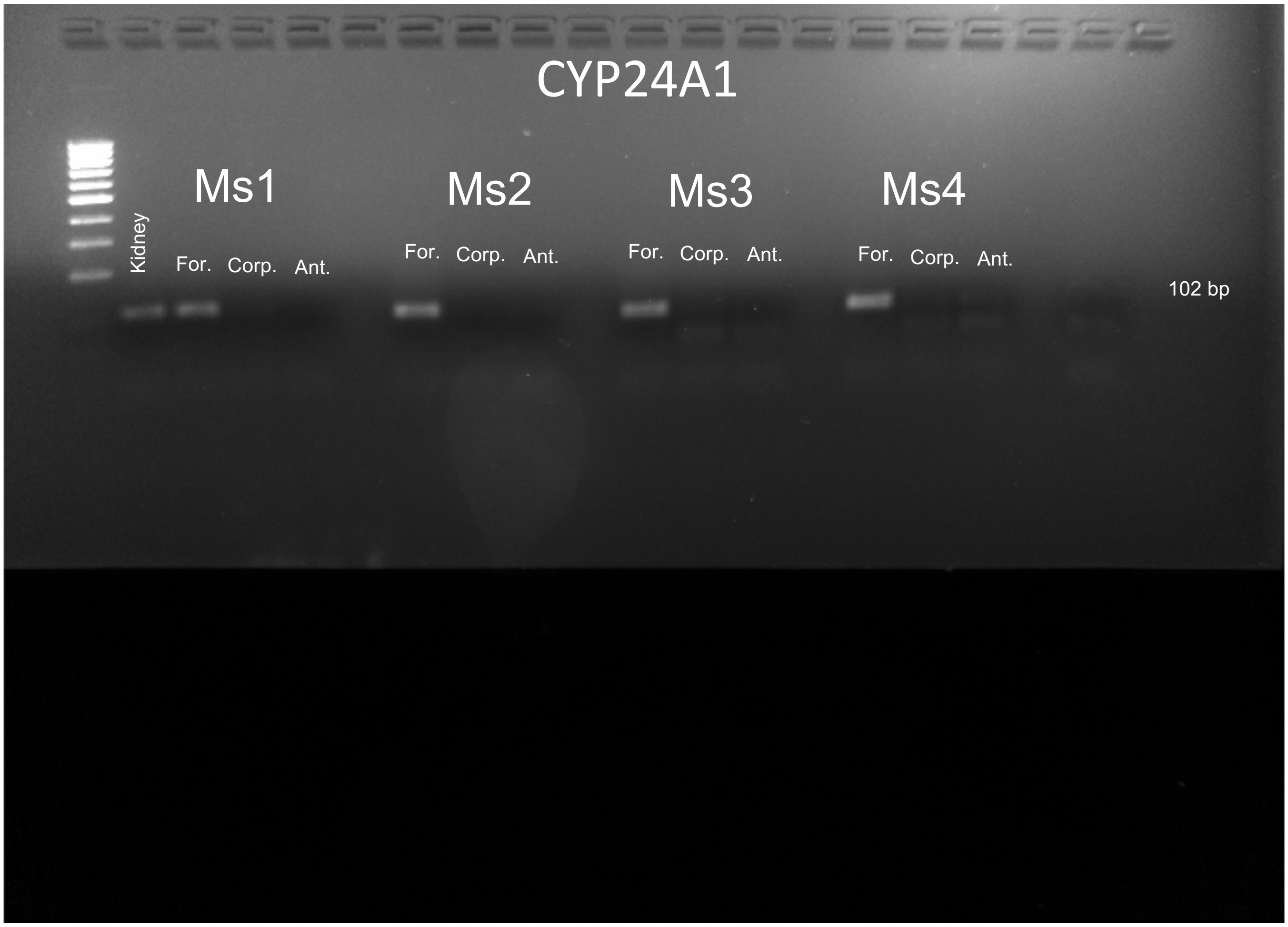

- Ladder used: 1000 , min is 50 bp

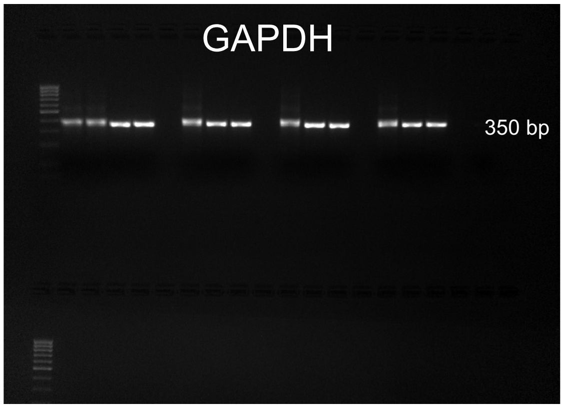

Supplement: Supplementary file 1 [file biology-10-00705-s001.zip › biology-1237266- file 1.pdf]
